# An association study in the Taiwan Biobank elicits three novel candidates for cognitive aging in old adults: *NCAM1, TTC12* and *ZBTB20*

**DOI:** 10.18632/aging.203321

**Published:** 2021-07-20

**Authors:** Eugene Lin, Po-Hsiu Kuo, Wan-Yu Lin, Yu-Li Liu, Albert C. Yang, Shih-Jen Tsai

**Affiliations:** 1 Department of Biostatistics, University of Washington, Seattle, WA 98195, USA; 2 Department of Electrical and Computer Engineering, University of Washington, Seattle, WA 98195, USA; 3 Graduate Institute of Biomedical Sciences, China Medical University, Taichung 40402, Taiwan; 4 Department of Public Health, Institute of Epidemiology and Preventive Medicine, National Taiwan University, Taipei 10617, Taiwan; 5 Department of Psychiatry, National Taiwan University Hospital, Taipei 100, Taiwan; 6 Center for Neuropsychiatric Research, National Health Research Institutes, Miaoli County 35053, Taiwan; 7 Division of Interdisciplinary Medicine and Biotechnology, Beth Israel Deaconess Medical Center, Harvard Medical School, Boston, MA 02215, USA; 8 Institute of Brain Science, National Yang Ming Chiao Tung University, Taipei 112304, Taiwan; 9 Department of Psychiatry, Taipei Veterans General Hospital, Taipei 11217, Taiwan; 10 Division of Psychiatry, National Yang Ming Chiao Tung University, Taipei 112304, Taiwan

**Keywords:** Alzheimer's diseases, cognitive aging, cognitive impairment, dopamine receptor, neurodegeneration

## Abstract

The dopamine receptor-related loci have been suggested to be associated with cognitive functions and neurodegenerative diseases. It is unknown whether genetic variants such as single nucleotide polymorphisms (SNPs) in the dopamine receptor-related loci could contribute to cognitive aging independently as well as by virtue of complicated interplays in the elder population. To assess whether SNPs in the dopamine receptor-related loci are associated with cognitive aging in the elder population, we evaluated SNPs in the *DRD1*, *NCAM1-TTC12-ANKK1-DRD2*, *DRD3-LOC107986115-ZNF80-TIGIT-MIR568-ZBTB20*, *DRD4*, and *DRD5-SLC2A9* loci from 25,195 older Taiwanese individuals from the Taiwan Biobank. Mini-Mental State Examination (MMSE) was scrutinized for all participants, where MMSE scores were employed to evaluate cognitive functions. From our analysis, we identified three novel genes for cognitive aging that have not previously been reported: *ZBTB20* on chromosome 3 and *NCAM1* and *TTC12* on chromosome 11. *NCAM1* and *ZBTB20* are strong candidates for having a role in cognitive aging with mutations in *ZBTB20* resulting in intellectual disability, and *NCAM1* previously found to be associated with associative memory in humans. Additionally, we found the effects of interplays between physical activity and these three novel genes. Our study suggests that genetic variants in the dopamine receptor-related loci may influence cognitive aging individually and by means of gene-physical activity interactions.

## INTRODUCTION

Dopamine is primarily recognized as a crucial factor for neurodegenerative diseases such as Alzheimer’s disease and Parkinson’s disease, which are normally characterized by gradual cognitive decline and dysfunction [1–3]. Furthermore, dopamine, mainly located in the midbrain, performs through five distinct subtypes of dopamine receptors (namely D1, D2, D3, D4, and D5 receptors) [4]. The dopamine receptors, G-protein coupled receptors, have been a focus of attention in the arena of drug design and discovery, where it is a target for drugs which treat various disorders such as Parkinson’s disease and psychiatric disorders [5–9].

The dopamine receptor genes *DRD1*, *DRD2*, *DRD3*, *DRD4*, and *DRD5* encode the D1, D2, D3, D4, and D5 subtype of the dopamine receptors, respectively. Genetic variants, in particular, single nucleotide polymorphisms (SNPs), have been described in the dopamine receptor gene research [10–12]. Literature suggests a connection between the dopamine receptor-related loci and neurodegenerative diseases, as well as between the dopamine receptor-related loci and cognitive functions in human beings [13]. For example, Tsang et al. [14] reported that the *DRD1* gene was associated with inferior cognitive performance in the postmortem cohort of Caucasian samples with Alzheimer's disease. Furthermore, it has been demonstrated that the *DRD2* gene was linked to better cognitive performance in verbal learning following traumatic brain injury [15] and may influence cognitive performance (such as number of categories achieved in the Wisconsin Card Sorting Test) in healthy individuals [16]. Mota et al. [17] also suggested that *DRD2* co-regulates with three nearby genes (*NCAM1*, *TTC12*, and *ANKK1*) to form the *NCAM1-TTC12-ANKK1-DRD2* locus which correlates with dopaminergic neurotransmission and neurogenesis. Moreover, Cordeddu et al. [18] found that *DRD3* joins with its adjacent genes (*LOC107986115*, *ZNF80*, *TIGIT*, *MIR568*, and *ZBTB20*) to constitute the *DRD3-LOC107986115-ZNF80-TIGIT-MIR568-ZBTB20* locus which contributes to Primrose syndrome, a genetic disorder with intellectual disability and learning difficulties. In addition, it has been indicated that the *DRD4* gene exhibits a significant association in Alzheimer’s disease in the Taiwanese population [19] and is linked to a specific cognitive domain called perceptual speed performance in cognitively healthy individuals of European ancestry [20]. Finally, Hollingworth et al. [21] identified the *SLC2A9* gene, which is neighboring to *DRD5*, as a susceptibility gene for psychosis and Alzheimer’s disease in populations of European ancestry.

In light of the aforementioned observations, it was presumed that the dopamine receptor-related loci, including the *DRD1*, *NCAM1-TTC12-ANKK1-DRD2*, *DRD3-LOC107986115-ZNF80-TIGIT-MIR568-ZBTB20*, *DRD4*, and *DRD5-SLC2A9* loci, may play a key role in the pathogenesis of age-dependent cognitive decline and the development of cognitive aging. Here, we hypothesized that genetic variants such as SNPs in the dopamine receptor-related loci might be associated with cognitive aging in the population with Taiwanese ancestry. To the best of our knowledge, the effects of genetic variants in the dopamine receptor-related loci on cognitive aging are scant in regards to human studies. In addition, several studies [22–27] in the Taiwan Biobank have conducted association analyses on cognitive aging using Mini-Mental State Examination (MMSE) scores, a standard method provided in the Taiwan Biobank. Based on the aforementioned considerations, we conducted the first genetic association study between MMSE scores and SNPs in the dopamine receptor-related loci in the Taiwan Biobank. We detected associations between MMSE scores and genetic variants in three genes (including *NCAM1* and *TTC12* on chromosome 11 and *ZBTB20* on chromosome 3) which have not been previously discovered. It has also been indicated that environmental factors such as physical activity are linked to cognitive aging as well [22–25]. Substantial evidence reveals that physical activity contributes to a lower risk of developing cognitive impairment and Alzheimer's disease [28]. Thus, we assessed the probable gene-physical activity interactions on cognitive aging and found potential gene-physical activity interactions with the *NCAM1, TTC12*, and *ZBTB20* genes in influencing cognitive aging.

## RESULTS

### The clinical and demographic characteristics of the study cohort

Table 1 illustrates the clinical and demographic characteristics of our study cohort, which consisted of 25,195 individuals from the Taiwan Biobank. The median MMSE score was 28 and the interquartile range was 26–29. Supplementary Table 1 presents the demographic characteristics and the relevant MMSE scores in our study cohort. In this study, we found that correlations between MMSE scores with age (P < 0.001), gender (P < 0.001), education (P < 0.001), physical activity (P < 0.001), smoking (P = 0.004), and chronic conditions (P < 0.001) were significant (Supplementary Table 1).

**Table 1:** Demographic and clinical characteristics of study subjects.

**Characteristic**	**Overall**
No. of subjects, n	25,195
Mean age ± SD, years	64.3±3.2
Female, %	61.2
Education level ^1^, % “1” for no formal education, “2” for homeschooling, “3” for elementary school, “4” for middle school, “5” for high school, "6" for college, and "7" for graduate school	1: 8.9 2: 14.3 3: 12.8 4: 9.0 5: 23.4 6: 27.2 7: 4.4
Physical activity, %	63.6
Smoking, %	5.6
Alcohol drinking, %	5.3
Chronic conditions, %	84.7
MMSE score, median (IQR)	28 (26–29)

IQR, interquartile range; MMSE, Mini-Mental State Examination; SD, standard deviation. Data are presented as mean ± standard deviation.

^1^Education level is defined as the following seven levels: no formal education, homeschooling, elementary school, middle school, high school, college, and graduate school.

### Association of cognitive aging in the dopamine receptor-related loci

First, we explored the associations between cognitive aging and the dopamine receptor-related loci, including the *DRD1*, *NCAM1-TTC12-ANKK1-DRD2*, *DRD3-LOC107986115-ZNF80-TIGIT-MIR568-ZBTB20*, *DRD4*, and *DRD5-SLC2A9* loci. Among the 791 SNPs investigated in the present study, there were 116 SNPs within the dopamine receptor-related loci revealing evidence of associations (P < 0.05) with MMSE scores (Supplementary Tables 2–6).

**Figure 1:**
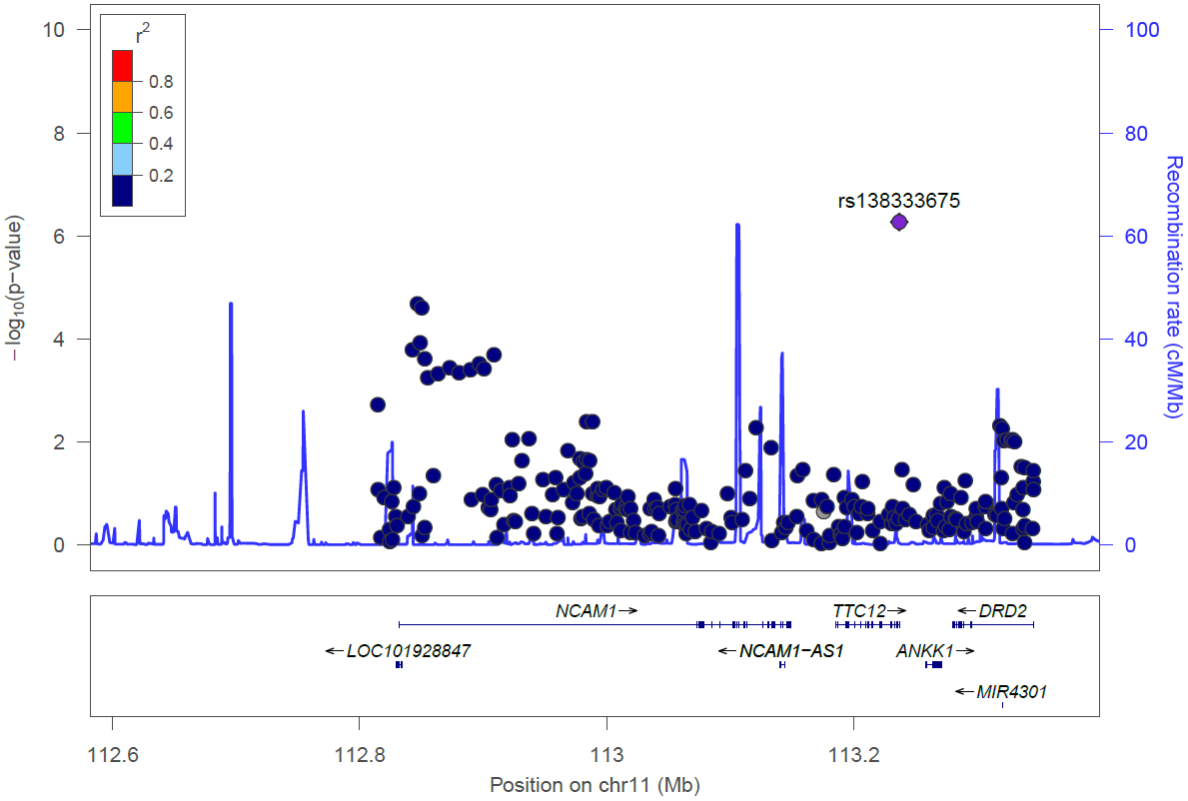
**Locus zoom plot for the *NCAM1-TTC12-ANKK1-DRD2* locus for cognitive aging in the Taiwan Biobank.** SNPs are shown by their position on the chromosome against their association (−log_10_ P) with cognitive aging. SNPs are colored to reflect their linkage disequilibrium with the top SNP (rs138333675) in *TTC12*. Estimated recombination rates are plotted in cyan using Asian subjects from the 1000 Genomes Project. This plot was generated using LocusZoom.

Figure 1 shows locus zoom plots illustrating the association results in the *NCAM1-TTC12-ANKK1-DRD2* locus. There were 46 SNPs in the *NCAM1-TTC12-ANKK1-DRD2* locus revealing evidence of associations (P < 0.05) with MMSE scores (Supplementary Table 2). As illustrated in Table 2, the significance persisted for the association of MMSE scores among 3 SNPs after employing Bonferroni correction (P < 0.05/791 = 6.32 x 10^-5^), including rs11214442 in *NCAM1* (P = 2.06 x 10^-5^), rs10891485 in *NCAM1* (P = 2.54 x 10^-5^), and rs138333675 in *TTC12* (P = 5.39 x 10^-7^). The two significant SNPs (rs11214442 and rs10891485) in *NCAM1* are not in LD with rs138333675 in *TTC12*, suggesting the signals from *NCAM1* and *TTC12* were independent (Supplementary Table 7). Moreover, these two SNPs (rs11214442 and rs10891485) in *NCAM1* are in LD with each other in the Taiwanese population, suggesting they are the same signal (Supplementary Table 7). Next, we investigated the likely roles of the above 3 SNPs as expression quantitative trait loci (eQTLs). We found that rs11214442 and rs10891485 in *NCAM1* is involved in regulating expressions of the *NCAM1* gene in artery/aorta tissues [29].

**Table 2:** Linear regression models of associations between the MMSE scores and 6 SNPs in the *NCAM1*, *TTC12*, and *ZBTB20* genes, which have an evidence of association (P < 0.05) and remain significant after employing Bonferroni correction (P < 0.05/791 = 6.32 x 10^-5^).

**CHR**	**Gene**	**SNP**	**A1**	**A2**	**Region**	**MAF**	**Dominant model**				**Recessive model**		
							**BETA**	**SE**	**P**		**BETA**	**SE**	**P**
3	*ZBTB20*	rs145272406	C	T	intron	0.015	-0.12	0.09	0.177		-4.27	0.99	**1.59 x 10^-5^**
3	*ZBTB20*	rs114295131	A	C	intron	0.015	-0.12	0.09	0.174		-4.27	0.99	**1.59 x 10^-5^**
3	*ZBTB20*	rs77949732	T	A	intron	0.015	-0.11	0.09	0.222		-4.27	0.99	**1.59 x 10^-5^**
11	*NCAM1*	rs11214442	G	A	intron	0.304	0.13	0.03	**2.06 x 10^-5^**		0.14	0.05	0.011
11	*NCAM1*	rs10891485	G	A	intron	0.303	0.13	0.03	**2.54 x 10^-5^**		0.13	0.05	0.015
11	*TTC12*	rs138333675	A	G	missense	0.029	-0.01	0.07	0.879		-2.54	0.51	**5.39 x 10^-7^**

A1, minor allele; A2, major allele; BETA, Beta coefficients; CHR, chromosome; MAF, minor allele frequency; MMSE, Mini-Mental State Examination; SE, standard error.

Analysis was obtained after adjustment for covariates including age, gender, education, physical activity, smoking, alcohol drinking, and chronic conditions.

P values of < 0.05/791 = 6.32 x 10^-5^ are shown in bold.

**Figure 2:**
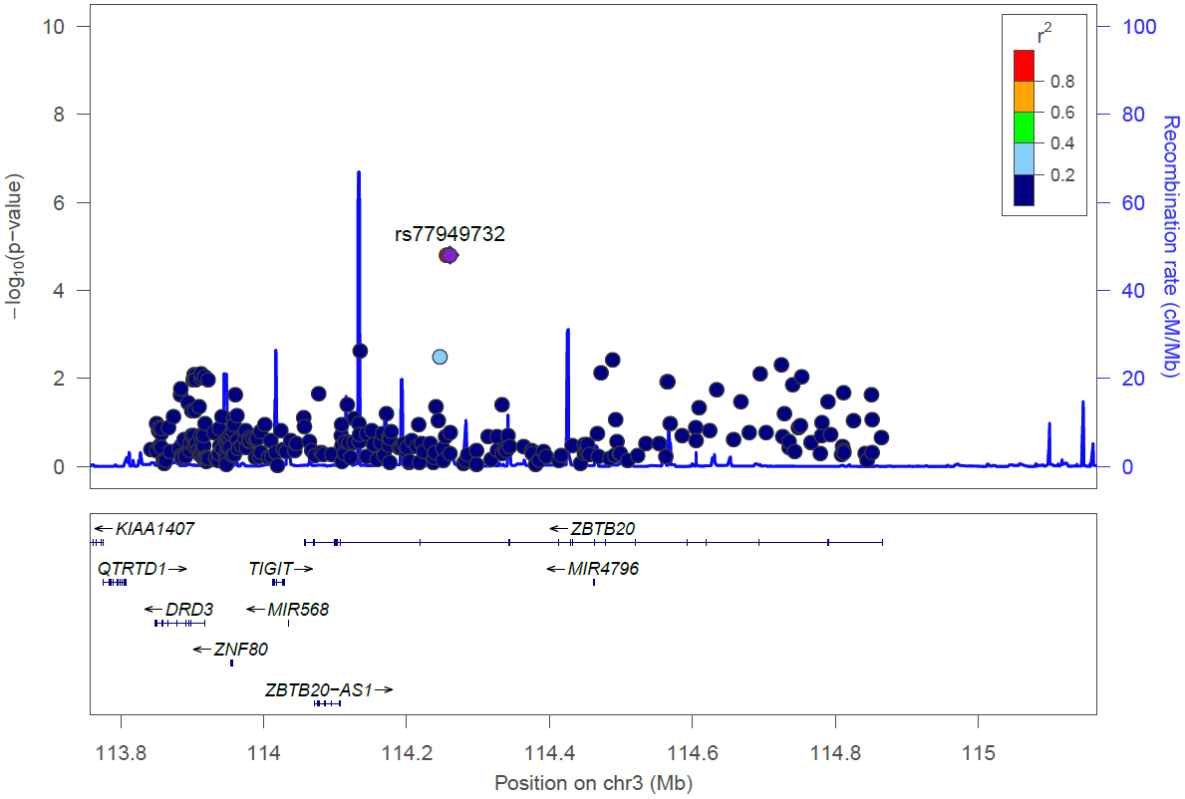
**Locus zoom plot for the *DRD3-LOC107986115-ZNF80-TIGIT-MIR568-ZBTB20* locus for cognitive aging in the Taiwan Biobank.** SNPs are shown by their position on the chromosome against their association (−log_10_ P) with cognitive aging. SNPs are colored to reflect their linkage disequilibrium with the top SNP (rs77949732) in *ZBTB20*. Estimated recombination rates are plotted in cyan using Asian subjects from the 1000 Genomes Project. This plot was generated using LocusZoom.

Figure 2 shows locus zoom plots illustrating the association results in the *DRD3-LOC107986115-ZNF80-TIGIT-MIR568-ZBTB20* locus. There were 44 SNPs in the *DRD3-LOC107986115-ZNF80-TIGIT-MIR568-ZBTB20* locus revealing evidence of associations (P < 0.05) with MMSE scores (Supplementary Table 3). As illustrated in Table 2, the significance persisted for the association of MMSE scores among 3 SNPs after employing Bonferroni correction (P < 0.05/791 = 6.32 x 10^-5^), including rs145272406 in *ZBTB20* (P = 1.59 x 10^-5^), rs114295131 in *ZBTB20* (P = 1.59 x 10^-5^), rs77949732 in *ZBTB20* (P = 1.59 x 10^-5^). The three significant SNPs (rs145272406, rs114295131, and rs77949732) in *ZBTB20* are in LD with each other in the Taiwanese population, suggesting they are the same signal (Supplementary Table 8). However, there was no potential mechanism for these 3 SNPs as eQTLs.

Supplementary Figures 1–3 show locus zoom plots illustrating the association results in the *DRD1*, *DRD4*, and *DRD5-SLC2A9* loci, respectively. There were 11 SNPs in the *DRD1* locus, 3 SNPs in the *DRD4* locus, and 12 SNPs in the *DRD5-SLC2A9* locus revealing evidence of associations (P < 0.05) with MMSE scores (Supplementary Tables 4–6). However, none of these SNPs in the *DRD1*, *DRD4*, and *DRD5-SLC2A9* loci remained significant after employing Bonferroni correction.

### Physical activity and gene interaction analysis

Next, we utilized categorized MMSE scores as an outcome (normal: MMSE score ≥ 24; cognitive impairment: MMSE score < 24) for physical activity and gene interaction analysis. The GMDR method was employed to estimate the effects of consolidation between physical activity and the three key SNPs (namely rs11214442 in *NCAM1*, rs138333675 in *TTC12*, and rs77949732 in *ZBTB20*) in cognitive aging by incorporating age, gender, and education as covariates. Here, we only selected one top SNP from each of *NCAM1*, *TTC12*, and *ZBTB20*. As illustrated in Table 3, there were significant two-way models concerning physical activity among rs11214442 in *NCAM1*, rs138333675 in *TTC12*, and rs77949732 in *ZBTB20* (P < 0.001, < 0.001, and < 0.001, respectively), indicating potential physical activity and gene interactions between these genes and physical activity in regulating cognitive aging. The effect of these physical activity and gene interaction models remained significant after Bonferroni correction (P < 0.05/3 = 0.017).

**Table 3:** Physical activity and gene interaction models identified by the GMDR method.

**2-way interaction model**	**Testing accuracy (%)**	**P value**
Physical activity, rs11214442 in *NCAM1*	53.86	**< 0.001**
Physical activity, rs138333675 in *TTC12*	53.86	**< 0.001**
Physical activity, rs77949732 in *ZBTB20*	53.94	**< 0.001**

GMDR, generalized multifactor dimensionality reduction. P value was based on 1,000 permutations.

Analysis was obtained after adjustment for covariates including age, gender, education, smoking, alcohol drinking, and chronic conditions.

P values of < 0.017 (Bonferroni correction: 0.05/3) are shown in bold.

## DISCUSSION

To our knowledge, this is the first study to date to determine whether the main impacts of SNPs in the dopamine receptor-related loci are significantly associated with the risk of cognitive aging on an individual-by-individual basis and by virtue of gene-physical activity interactions in elder subjects. Here, we observed for the first time that the dopamine receptor-related loci may play an essential role in influencing cognitive aging in elder Taiwanese individuals. Intriguingly, the significance persisted for the association of MMSE scores with six key SNPs after correcting for multiple testing, including rs11214442 in *NCAM1*, rs10891485 in *NCAM1*, and rs138333675 in *TTC12*, rs145272406 in *ZBTB20*, rs114295131 in *ZBTB20*, and rs77949732 in *ZBTB20*. Moreover, the findings indicated that gene-physical activity interactions in the *NCAM1*, *TTC12*, and *ZBTB20* genes may modulate the etiology of cognitive aging.

Remarkably, the present study is the first to raise the possibility that three significant SNPs in the *NCAM1-TTC12-ANKK1-DRD2* locus, such as rs11214442 in *NCAM1*, rs10891485 in *NCAM1*, and rs138333675 in *TTC12*, may be associated with cognitive aging. Furthermore, the significant association between these key SNPs and MMSE scores persisted even after applying Bonferroni correction. We also found that rs11214442 and rs10891485 in *NCAM1* may play a functional role as eQTLs for the *NCAM1* gene in artery/aorta tissues [29]. To our knowledge, no other studies have been conducted to pinpoint these three SNPs in *NCAM1* and *TTC12* with cognitive aging or age-related cognitive decline. Both the *NCAM1* and *TTC12* genes, located on chromosome 11q23.2, are good candidates for cognitive aging because they represent the *NCAM1-TTC12-ANKK1-DRD2* locus which is linked to dopaminergic neurotransmission and neurogenesis [17]. It has also been indicated that *NCAM1* may play a key role in impairment of spatial memory in epileptic rats [30] as well as in associative memory in nematodes and humans [31]. Moreover, it has been suggested that the *NCAM1-TTC12-ANKK1-DRD2* locus should be considered as one single unit for investigating genetics in behavioral and psychiatric studies [17]. In addition, it has been reported that the *NCAM1-TTC12-ANKK1-DRD2* locus is considered as a risk biomarker for alcohol dependence [32], nicotine dependence [33], heroin dependence [34], and comorbid alcohol and drug dependence [35].

Interestingly, the present study is the first to raise the possibility that three significant SNPs in the *DRD3-LOC107986115-ZNF80-TIGIT-MIR568-ZBTB20* locus, such as rs145272406 in *ZBTB20*, rs114295131 in *ZBTB20*, and rs77949732 in *ZBTB20*, may be associated with cognitive aging. To our knowledge, no other studies have been conducted to pinpoint these three SNPs in *ZBTB20* with cognitive aging or age-related cognitive decline. The *ZBTB20* gene, located on chromosome 3q13.31, represents the *DRD3-LOC107986115-ZNF80-TIGIT-MIR568-ZBTB20* locus. *ZBTB20* is a good candidate for cognitive aging. For example, it has been reported that mutations in *ZBTB20* are associated with Primrose syndrome, a genetic disorder characterized by intellectual disability, autism, and other behavioral concerns [18]. On another note, *ZBTB20* has been previously implicated in influencing neurodevelopmental disorders in subjects of European ancestry [36]. Animal studies also revealed that the *Zbtb20* gene is mainly expressed in brain and its encoded protein is implicated in hippocampal development and cerebellar granule cells [37, 38].

Intriguingly, we pinpointed the interplays between the dopamine receptor-related loci and physical activity in affecting cognitive aging, where the dopamine receptor-related loci encompass *NCAM1*, *TTC12*, and *ZBTB20*. This relationship could functionally manifest itself on the basis of epigenetic alterations [23]. Our findings are in line with other association studies in the Taiwanese population from the Taiwan Biobank, indicating that physical activity may modulate cognitive aging by means of likely complex gene-physical activity interplays with the interleukin-12 related genes (such as *IL12A*, *IL12B*, and *IL12RB2*) [23], the DNA repair gene *EXO1* [24], circadian clock genes (such as *RORA* and *RORB*) [22], and Alzheimer's disease-associated genes (such as *SLC24A4*) [25]. Papenberg et al. [39] also suggested the detrimental effects of inflammation on cognitive aging in old adults who lack of physical activity. Furthermore, the dopamine receptor-related loci, such as *DRD2* and *DRD3*, have been found to play a central role in inflammation, neuroinflammation, and neurodegeneration [40, 41]. Thus, it is plausible to hypothesize that the interplays found in this study may be linked to the negative effects of inflammation on cognitive aging in inactive old adults.

In our analysis, there was also evidence of associations (P < 0.05) between MMSE scores and other dopamine receptor-related loci, such as *DRD1*, *ARL2BPP6-DRD1*, *DRD1-SFXN1*, *DRD2*, *NCAM1-LOC105369498*, *DRD3*, *DRD3-LOC107986115*, *ZNF80-TIGIT*, *DRD4-DEAF1*, and *SLC2A9*. The *DRD1*, *ARL2BPP6-DRD1*, and *DRD1-SFXN1* loci are located on chromosome 5q35.2. The *DRD2* and *NCAM1-LOC105369498* loci, located on chromosome 11q23.2, represent the *NCAM1-TTC12-ANKK1-DRD2* locus. The *DRD3*, *DRD3-LOC107986115*, and *ZNF80-TIGIT* loci, located on chromosome 3q13.31, represents the *DRD3-LOC107986115-ZNF80-TIGIT-MIR568-ZBTB20* locus. The *DRD4-DEAF1* locus is located on chromosome 11p15.5. The *SLC2A9* gene, located on chromosome 4p16.1, represents the *DRD5-SLC2A9* locus. In accordance with our results, it has been reported that *DRD1* [14], *DRD2* [15, 16], *DRD3* [18], *DRD4* [19, 20], and *SLC2A9* [21] are associated with cognitive performance or cognitive impairment.

This study had some limitations. First, the age effect was limited due to a homogeneous cohort in terms of age (i.e., the mean age of 64.3 years and the standard deviation of 3.2 years). Second, studies on cognitive aging require repetitive cognitive assessments of each individual over years and our study is limited to a single cognitive assessment of each individual. Furthermore, in our statistical analyses, adjustments were not made for other risk factors, namely brain injury and exposure to pesticides/toxins, due to a lack of such data. Future studies making use of multi-omics data are also warranted to provide the molecular mechanisms underlying cognitive aging.

In conclusion, the present study accomplished a thorough investigation of the associations of cognitive aging with the dopamine receptor-related loci in old adults in the Taiwanese population. Moreover, the present study revealed the impacts of gene-physical activity interactions in the dopamine receptor-related loci with regard to cognitive aging. In particular, if the current findings are reproduced in statistically well-powered distinct samples, the present study pinpoints the effects of the dopamine receptor-related loci on the risk of cognitive aging on an individual-by-individual basis as well as via sophisticated gene-physical activity interplays. The present study implicates that dopamine receptor-mediated signaling should be the focus of future studies on pathogenesis of age-dependent cognitive decline and probable drug targets for drug design and discovery. Distinct studies with greater replication datasets will potentially create further insights into the role of the dopamine receptor-related loci demonstrated in the present study.

## MATERIALS AND METHODS

### Study population

Our study cohort composed of 25,195 subjects for subsequent analyses. This study involved Taiwanese individuals from the Taiwan Biobank, which collected specimens and relevant information from participants in recruitment centers across Taiwan [22, 25–27, 42, 43]. There were the following two inclusion criteria: (1) participants who were 60 years old or over; and (2) participants who were self-reported as being of Taiwanese ancestry [43]. The exclusion criterion was participants with a history of cancer [43]. Ethical approval for the study was granted by the Institutional Review Board of the Taiwan Biobank before performing the study (approval number: 201506095RINC). The approved informed consent form was signed by each subject. All experiments were achieved by means of proper regulations and guidelines.

A subject’s education level includes the following seven levels: no formal education, homeschooling, elementary school, middle school, high school, college, and graduate school [22–27]. A subject who had exercised for over 30 minutes each time and over three times each week was defined as a measure of physical activity [22–25]. A subject with current smoking for more than 6 months was defined as a current smoker [22–25]. A subject with a volume of 150mL of alcohol intake per week for more than 6 months was defined as a current alcohol drinker [22–25]. Status of chronic conditions was defined as whether a subject or a subject's family member (i.e., family history) has had the following chronic conditions: Parkinson's disease, heart disease, stroke, and/or diabetes.

### Cognitive assessment

We utilized the 30-point MMSE, a cognitive impairment screening tool in the Taiwan Biobank, as previously described [22–27]. In brief, we assessed global cognitive performance by employing the 30-point MMSE, which encompasses questions in accordance with the five areas of recall, registration, language, attention and calculation, and orientation [27]. We assessed the MMSE score both as a continuous phenotype and as a binary phenotype in accordance with the following previously-established MMSE thresholds [44]: MMSE score ≥ 24 (normal) and MMSE score < 24 (cognitive impairment). The cognitive assessment was carried out in the local languages (such as Taiwanese and/or Taiwanese Mandarin). The cognitive cut-off score of 24 was originated from previous studies [44] and was based on a Taiwanese version of MMSE.

### Laboratory assessments: genotyping

DNA was extracted from blood samples by employing QIAamp DNA blood kits following the manufacturer’s instructions (Qiagen, Valencia, CA, USA). The quality of the isolated genomic DNA was carried out by utilizing agarose gel electrophoresis, and the quantity was completed by spectrophotometry [45]. SNP genotyping was evaluated by employing the custom Taiwan Biobank chips, which were accomplished by using the Axiom Genome-Wide Array Plate System (Affymetrix, Santa Clara, CA, USA). The custom Taiwan Biobank chips were created to collect genetic profiles in Taiwanese subjects by utilizing SNPs on the Axiom Genome-Wide CHB 1 Array (Affymetrix, Santa Clara, CA, USA) and the Human Exome BeadChip (Illumina, Inc., San Diego, CA, USA) [43].

We searched for variants in the *DRD1*, *NCAM1-TTC12-ANKK1-DRD2*, *DRD3-LOC107986115-ZNF80-TIGIT-MIR568-ZBTB20*, *DRD4*, and *DRD5-SLC2A9* loci by referring to the complete list of SNPs available in the custom Taiwan Biobank chips. In addition, we performed quality control procedures for subsequent analysis [46, 47]. The quality control procedure excluded troublesome SNPs that were not in Hardy-Weinberg equilibrium (HWE) (with a P-value less than 0.05) or had a genotyping call rate less than 95% or minor allele frequency (MAF) < 1%. We evaluated MAFs, genotyping call rates, P values for HWE using PLINK [48]. After the quality control procedure, the SNP panel consisted of 792 SNPs in the *DRD1*, *NCAM1-TTC12-ANKK1-DRD2*, *DRD3-LOC107986115-ZNF80-TIGIT-MIR568-ZBTB20*, *DRD4*, and *DRD5-SLC2A9* loci.

### Statistical analysis

The Student’s t test was performed to measure the difference in the means of two continuous variables [23]. We conducted the chi-square test for categorical data. The criterion for significance was set at P < 0.05 for all tests. Data are presented as the mean ± standard deviation.

In this study, linear regression analysis was carried out to evaluate the relationship between MMSE scores and our variables of interest such as age, gender, education, physical activity, smoking, alcohol drinking, and chronic conditions. In addition, we assessed the association of the investigated SNP with MMSE scores by a general linear model using age, gender, education, physical activity, smoking, alcohol drinking, and chronic conditions as covariates [49]. The genotype frequencies were performed for Hardy-Weinberg equilibrium to detect genotyping errors [50] by employing a χ^2^ goodness-of-fit test with one degree of freedom (that is, the number of genotypes minus the number of alleles). Adjustments for multiple testing were conducted by employing the Bonferroni correction. The criterion for significance was defined as P < 0.05 for all tests. Data were shown by the mean ± standard deviation.

In order to examine gene-physical activity interplays, we utilized the generalized multifactor dimensionality reduction (GMDR) method [51]. We scrutinized two-way interactions by employing 10-fold cross-validation. The GMDR method provided various output parameters, such as empirical P values and the testing accuracy, to evaluate each interaction. Furthermore, covariates such as age, gender, education, smoking, alcohol drinking, and chronic conditions were utilized for gene-physical activity interaction analysis in our interaction models. We completed the empirical P value of the testing accuracy for each interaction by employing permutation testing (based on 1,000 shuffles). Finally, we utilized the Bonferroni correction to correct for multiple testing.

In addition, we employed HaploReg (http://compbio.mit.edu/HaploReg) [52] to test if there is a functional role as eQTLs for the SNPs in the specific genes.

## Supplementary Material

Supplementary Figures

Supplementary Tables
