# Supplementary material for: An association study in the Taiwan Biobank elicits three novel candidates for cognitive aging in old adults: NCAM1, TTC12 and ZBTB20
Source: Aging (Albany NY). 2021 Jul 20;13(14):18769–88. doi: 10.18632/aging.203321 (PMC8351692; doi:10.18632/aging.203321)
Supplement: Supplementary Figures [file aging-13-203321-s001.pdf]

SUPPLEMENTARY FIGURES

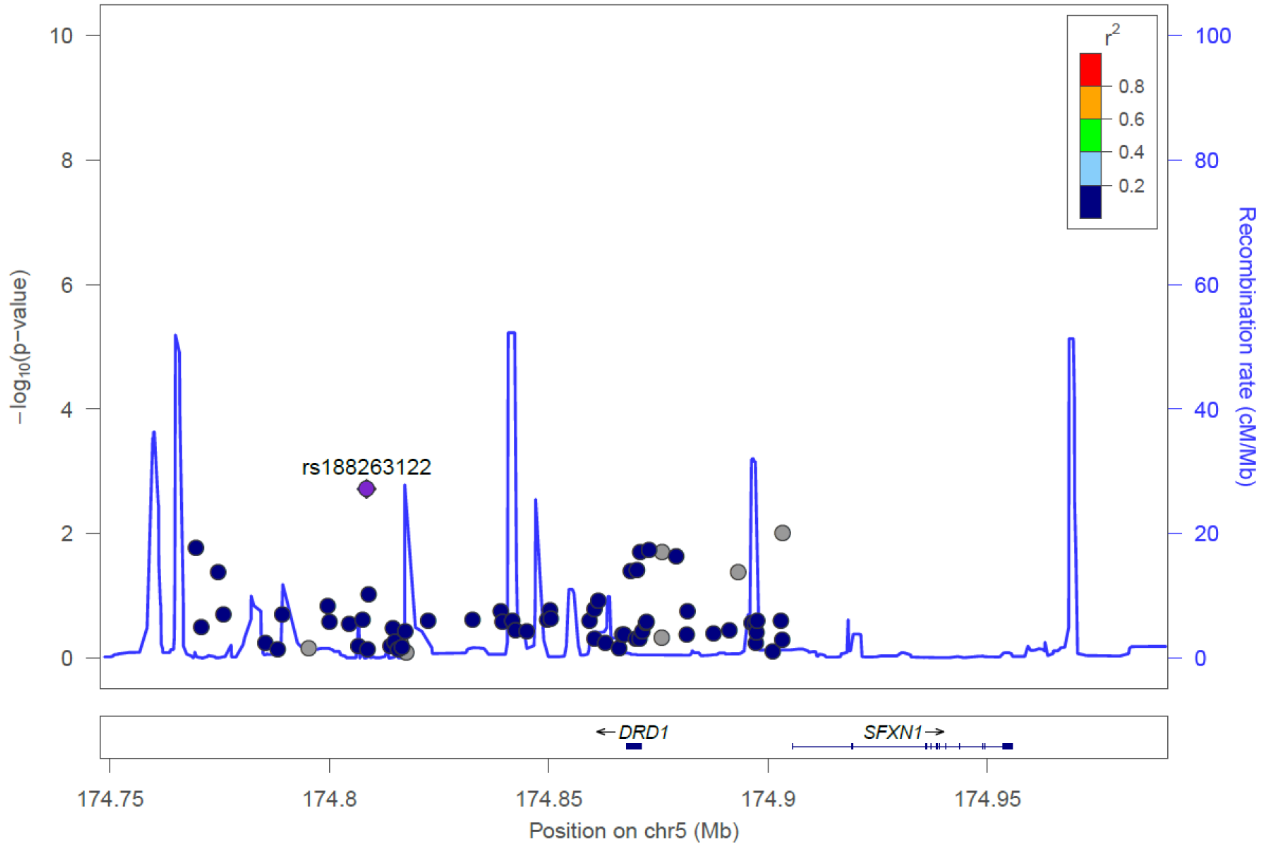

Supplementary Figure 1. Locus zoom plot for the *DRD1* locus for cognitive aging in the Taiwan Biobank. SNPs are shown by their position on the chromosome against their association ($-\log_{10}$ P) with cognitive aging. SNPs are colored to reflect their linkage disequilibrium with the top SNP (rs188263122) near *DRD1* (*ARL2BPP6-DRD1*). Estimated recombination rates are plotted in cyan using Asian subjects from the 1000 Genomes Project. This plot was generated using LocusZoom.

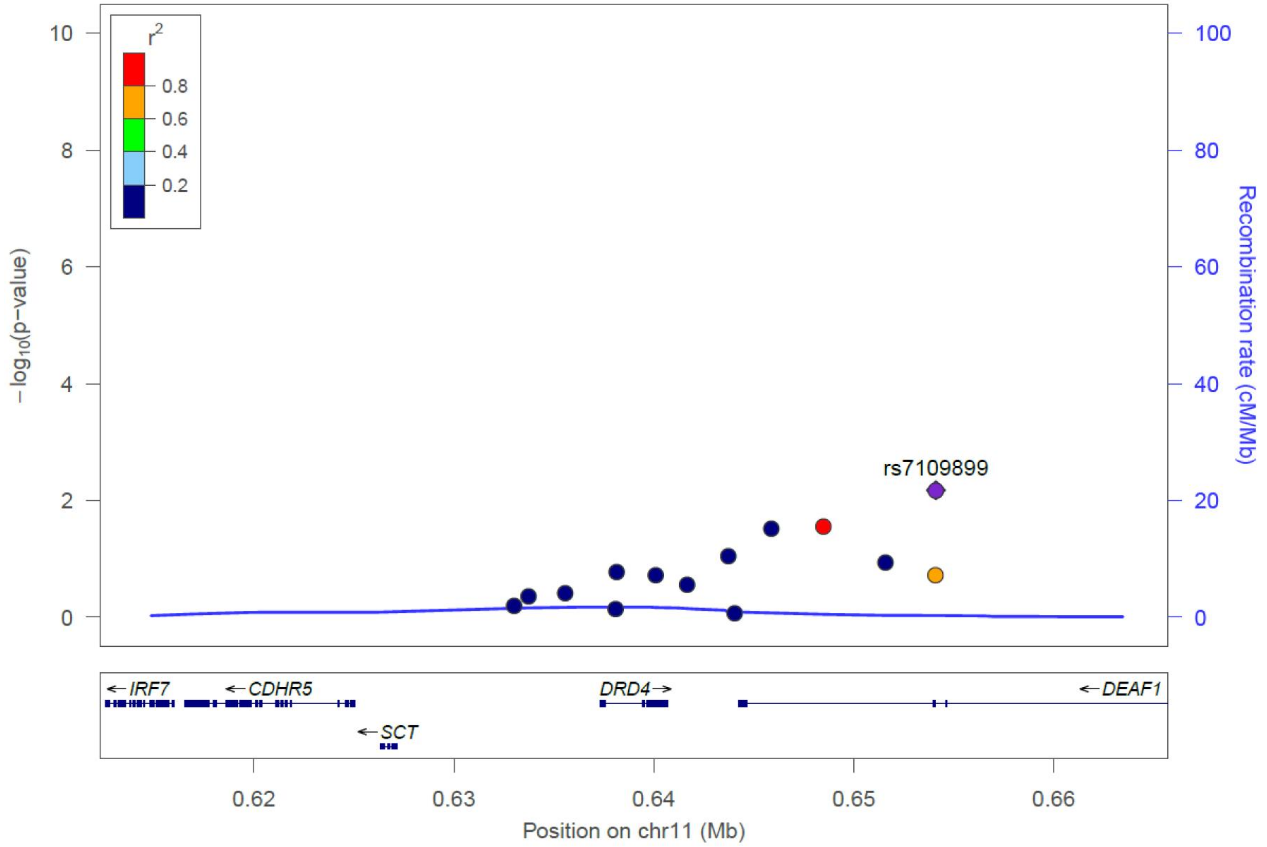

Supplementary Figure 2. Locus zoom plot for the *DRD4* locus for cognitive aging in the Taiwan Biobank. SNPs are shown by their position on the chromosome against their association ($-\log_{10}$ P) with cognitive aging. SNPs are colored to reflect their linkage disequilibrium with the top SNP (rs7109899) near *DRD4* (*DRD4-DEAF1*). Estimated recombination rates are plotted in cyan using Asian subjects from the 1000 Genomes Project. This plot was generated using LocusZoom.

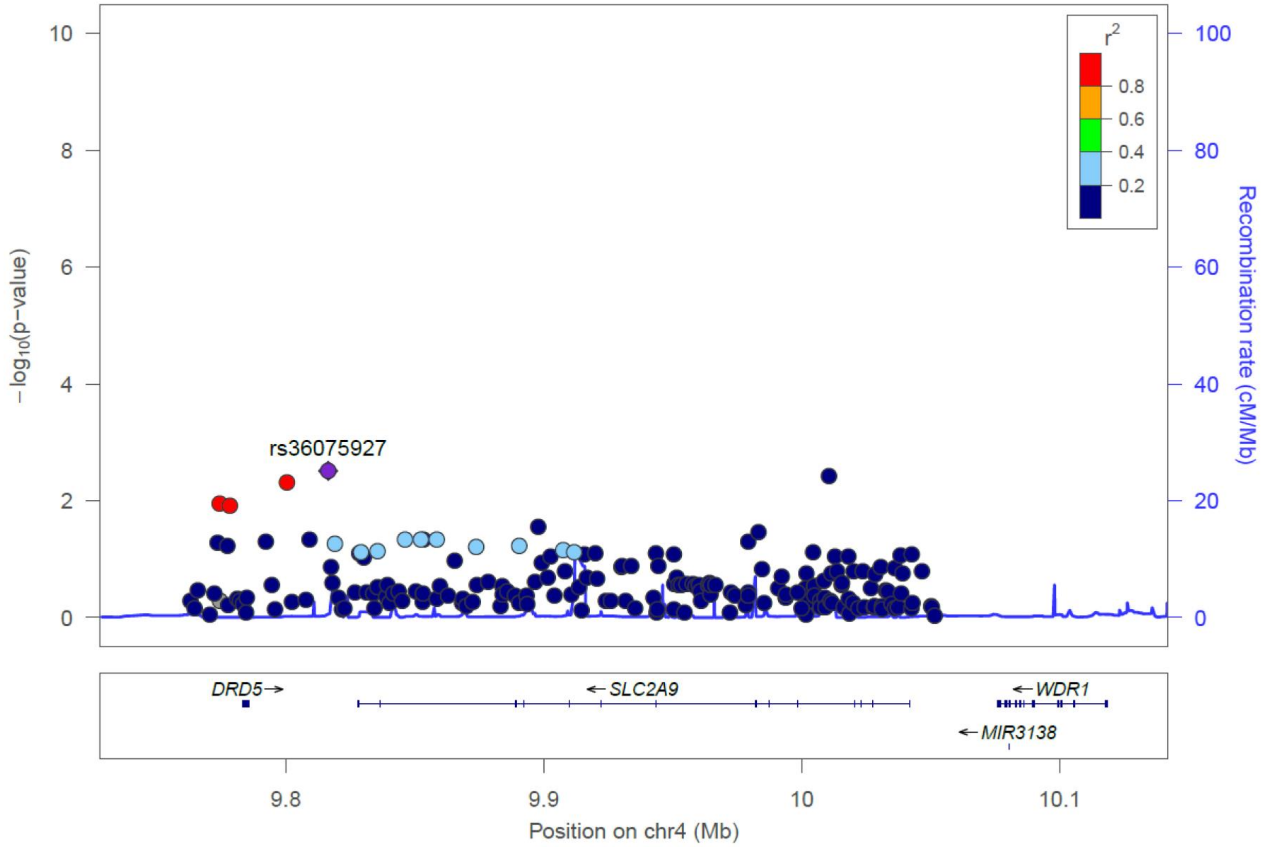

Supplementary Figure 3. Locus zoom plot for the *DRD5-SLC2A9* locus for cognitive aging in the Taiwan Biobank. SNPs are shown by their position on the chromosome against their association (−$\log_{10}$ P) with cognitive aging. SNPs are colored to reflect their linkage disequilibrium with the top SNP (rs36075927) in *SLC2A9*. Estimated recombination rates are plotted in cyan using Asian subjects from the 1000 Genomes Project. This plot was generated using LocusZoom.
