# Supplementary material for: An association study in the Taiwan Biobank elicits three novel candidates for cognitive aging in old adults: NCAM1, TTC12 and ZBTB20
Source: Aging (Albany NY). 2021 Jul 20;13(14):18769–88. doi: 10.18632/aging.203321 (PMC8351692; doi:10.18632/aging.203321)
Supplement: Supplementary Tables [file aging-13-203321-s002.pdf]

SUPPLEMENTARY TABLES

Supplementary Table 1. The demographic characteristics and MMSE scores of study subjects.

Characteristic	MMSE score (Mean ± SD)	P
Gender		
Male	27.5±2.3	< 0.001
Female	27.3±2.6	
Education level [1]		
"1" for no formal education,	1: 27.1±3.1	< 0.001
"2" for homeschooling,	2: 27.4±2.4	
"3" for elementary school,	3: 25.2±3.2	
"4" for middle school,	4: 26.7±2.5	
"5" for high school,	5: 27.7±1.9	
"6" for college, and	6: 28.2±1.7	
"7" for graduate school	7: 28.5±1.6	
Physical activity		
Yes	27.5±2.4	< 0.001
No	27.2±2.6	
Smoking		
Yes	27.2±2.6	0.004
No	27.4±2.5	
Alcohol drinking		
Yes	27.4±2.3	0.279
No	27.4±2.5	
Chronic conditions		
Yes	27.4±2.4	< 0.001
No	27.0±2.8	

MMSE, Mini-Mental State Examination; SD, standard deviation.
Data are presented as mean ± standard deviation.
[1]Education level is defined as the following seven levels: no formal education, homeschooling, elementary school, middle school, high school, college, and graduate school.

Supplementary Table 2. Linear regression models of associations between the MMSE scores and 46 SNPs in the *NCAM1-TTC12-ANKK1-DRD2* locus, which have an evidence of association (P < 0.05).

CHR	Gene	SNP	A1	A2	Region	MAF	Dominant model			Recessive model		
							BETA	SE	P	BETA	SE	P
11	*NCAM1-LOC105369498*	rs143926817	G	A	intergenic	0.016	-0.27	0.09	**1.94E-03**	-0.27	1.08	0.806
11	*NCAM1*	rs10789931	T	C	intron	0.468	-0.06	0.03	0.079	-0.14	0.04	**1.62E-04**
11	*NCAM1*	rs11214442	G	A	intron	0.304	0.13	0.03	**2.06E-05**	0.14	0.05	**0.011**
11	*NCAM1*	rs61905366	G	A	intron	0.312	0.12	0.03	**1.20E-04**	0.14	0.05	**6.61E-03**
11	*NCAM1*	rs10891485	G	A	intron	0.303	0.13	0.03	**2.54E-05**	0.13	0.05	**0.015**
11	*NCAM1*	rs1940725	T	C	intron	0.182	0.12	0.03	**2.45E-04**	0.04	0.09	0.627
11	*NCAM1*	rs7128707	T	A	intron	0.443	0.11	0.03	**5.81E-04**	0.09	0.04	**0.020**
11	*NCAM1*	rs1940717	A	G	intron	0.121	0.07	0.04	0.070	0.26	0.13	**0.045**
11	*NCAM1*	rs4424705	T	A	intron	0.443	0.12	0.03	**4.74E-04**	0.09	0.04	**0.020**
11	*NCAM1*	rs12363408	A	C	intron	0.444	0.12	0.03	**3.68E-04**	0.09	0.04	**0.021**
11	*NCAM1*	rs1884	G	C	intron	0.443	0.12	0.03	**4.55E-04**	0.09	0.04	**0.019**
11	*NCAM1*	rs61902793	G	A	intron	0.444	0.12	0.03	**3.92E-04**	0.09	0.04	**0.018**
11	*NCAM1*	rs11214468	C	T	intron	0.444	0.12	0.03	**3.12E-04**	0.09	0.04	**0.015**
11	*NCAM1*	rs12365502	C	A	intron	0.444	0.12	0.03	**3.76E-04**	0.09	0.04	**0.015**
11	*NCAM1*	rs1940735	T	G	intron	0.440	0.12	0.03	**2.00E-04**	0.09	0.04	**0.024**
11	*NCAM1*	rs10750023	A	G	intron	0.080	0.11	0.04	**9.25E-03**	-0.04	0.20	0.858
11	*NCAM1*	rs1836799	C	T	intron	0.374	0.07	0.03	**0.023**	0.00	0.04	0.911
11	*NCAM1*	rs1245089	C	G	intron	0.081	0.11	0.04	**8.62E-03**	-0.05	0.19	0.790
11	*NCAM1*	rs1816537	C	A	intron	0.091	0.10	0.04	**0.015**	0.25	0.18	0.169
11	*NCAM1*	rs12788208	A	G	intron	0.091	0.09	0.04	**0.021**	0.25	0.18	0.155

11	*NCAM1*	rs56268408	T	C	intron	0.099	0.09	0.04	**0.024**	-0.13	0.16	0.415
11	*NCAM1*	rs189464800	G	A	intron	0.023	-0.15	0.07	**0.042**	0.12	0.73	0.868
11	*NCAM1*	rs10891508	A	G	intron	0.376	-0.06	0.03	0.064	-0.13	0.04	**4.10E-03**
11	*NCAM1*	rs72995965	T	A	intron	0.100	0.09	0.04	**0.022**	-0.11	0.16	0.513
11	*NCAM1*	rs873803	A	G	intron	0.139	-0.08	0.04	**0.031**	-0.26	0.11	**0.023**
11	*NCAM1*	rs10891509	G	T	intron	0.376	-0.06	0.03	0.063	-0.13	0.04	**4.15E-03**
11	*NCAM1*	rs595231	C	T	intron	0.391	-0.08	0.03	**0.016**	-0.05	0.04	0.279
11	*NCAM1*	rs144186126	T	G	intron	0.022	-0.07	0.08	0.333	-1.70	0.81	**0.036**
11	*NCAM1*	rs146103467	T	C	intron	0.011	-0.08	0.10	0.446	-4.76	1.71	**5.38E-03**
11	*NCAM1*	rs146627958	G	A	intron	0.022	-0.19	0.08	**0.013**	1.60	0.73	**0.029**
11	*NCAM1-TTC12*	rs185398311	T	C	intergenic	0.012	-0.21	0.10	**0.045**	-1.83	2.43	0.450
11	*NCAM1-TTC12*	rs56159811	T	C	intergenic	0.056	-0.05	0.05	0.314	-0.56	0.27	**0.034**
11	*TTC12*	rs186496476	A	G	2KB upstream	0.016	-0.18	0.09	**0.043**	1.90	1.40	0.173
11	*TTC12*	rs138333675	A	G	missense	0.029	-0.01	0.07	0.879	-2.54	0.51	**5.39E-07**
11	*TTC12*	rs184198959	T	C	missense	0.013	-0.21	0.10	**0.034**	-1.83	2.42	0.450
11	*DRD2*	rs4936270	T	C	intron	0.137	0.00	0.04	0.894	-0.32	0.11	**4.78E-03**
11	*DRD2*	rs4274224	G	A	intron	0.167	-0.01	0.03	0.750	-0.18	0.09	**0.049**
11	*DRD2*	rs4245148	T	C	intron	0.137	0.00	0.04	0.993	-0.31	0.11	**5.66E-03**
11	*DRD2*	rs4460839	C	T	intron	0.137	0.00	0.04	0.966	-0.29	0.11	**9.32E-03**
11	*DRD2*	rs4581480	C	T	intron	0.137	0.00	0.04	0.992	-0.29	0.11	**9.08E-03**
11	*DRD2*	rs7122454	C	G	intron	0.393	0.08	0.03	**9.57E-03**	0.03	0.04	0.553
11	*DRD2*	rs10891550	C	G	intron	0.393	0.08	0.03	**9.11E-03**	0.03	0.04	0.476
11	*DRD2*	rs11214611	G	A	intron	0.393	0.08	0.03	**9.95E-03**	0.03	0.04	0.468
11	*DRD2*	rs4350392	A	C	intron	0.377	0.07	0.03	**0.030**	0.02	0.04	0.639
11	*DRD2*	rs4245149	A	G	intron	0.377	0.07	0.03	**0.032**	0.02	0.04	0.646
11	*DRD2*	rs10789944	A	C	intron	0.377	0.07	0.03	**0.036**	0.02	0.04	0.668

A1, minor allele; A2, major allele; CHR, chromosome; BETA, Beta coefficients; MAF, minor allele frequency; MMSE, Mini-Mental State Examination; SE, standard error.

Analysis was obtained after adjustment for covariates including age, gender, education, physical activity, smoking, alcohol drinking, and chronic conditions.

P values of < 0.05 are shown in bold.

Supplementary Table 3. Linear regression models of associations between the MMSE scores and 44 SNPs in the *DRD3-LOC107986115-ZNF80-TIGIT-MIR568-ZBTB20* locus, which have an evidence of association (P < 0.05).

CHR	Gene	SNP	A1	A2	Region	MAF	Dominant model			Recessive model		
							BETA	SE	P	BETA	SE	P
3	*DRD3*	rs144005981	A	G	intron	0.016	0.02	0.09	0.782	-2.74	1.21	0.024
3	*DRD3*	rs2971569	G	T	intron	0.386	0.08	0.03	**0.017**	0.05	0.04	0.262
3	*DRD3*	rs7638876	C	T	intron	0.341	0.07	0.03	**0.035**	0.07	0.05	0.161
3	*DRD3*	rs62267154	C	G	intron	0.252	0.08	0.03	**0.011**	0.07	0.06	0.238
3	*DRD3*	rs36212178	G	A	intron	0.238	0.08	0.03	**8.61E-03**	0.08	0.07	0.218
3	*DRD3*	rs1587756	C	T	intron	0.252	0.08	0.03	**0.011**	0.07	0.06	0.238
3	*DRD3*	rs1587757	G	A	intron	0.238	0.08	0.03	**8.46E-03**	0.08	0.07	0.210
3	*DRD3*	rs1587758	G	A	intron	0.252	0.08	0.03	**0.010**	0.07	0.06	0.230
3	*DRD3*	rs36212515	C	T	intron	0.251	0.08	0.03	**8.44E-03**	0.08	0.06	0.199
3	*DRD3*	rs12633630	C	A	intron	0.261	0.08	0.03	**8.65E-03**	0.06	0.06	0.304
3	*DRD3*	rs56362817	A	C	intron	0.252	0.08	0.03	**0.010**	0.08	0.06	0.228
3	*DRD3*	rs55953427	A	G	intron	0.252	0.08	0.03	**0.011**	0.08	0.06	0.223
3	*DRD3*	rs6804925	T	A	intron	0.252	0.08	0.03	**0.011**	0.08	0.06	0.223
3	*DRD3*	rs1394016	A	G	intron	0.356	0.06	0.03	**0.043**	0.08	0.05	0.066
3	*DRD3*	rs17605608	A	G	intron	0.261	0.08	0.03	**9.83E-03**	0.06	0.06	0.299
3	*DRD3*	rs57504078	G	A	intron	0.238	0.08	0.03	**7.79E-03**	0.08	0.07	0.209
3	*DRD3*	rs62268962	T	A	intron	0.262	0.08	0.03	**9.38E-03**	0.05	0.06	0.391
3	*DRD3-LOC107986115*	rs7649438	G	C	intergenic	0.262	0.08	0.03	**0.011**	0.06	0.06	0.343
3	*ZNF80-TIGIT*	rs78899496	A	G	intergenic	0.026	0.16	0.07	**0.023**	-0.26	0.52	0.609
3	*ZNF80-TIGIT*	rs965970177	T	G	intergenic	0.010	-0.09	0.11	0.431	-7.93	2.42	**1.07E-03**
3	*ZBTB20*	rs75995250	A	G	intron	0.086	0.09	0.04	**0.042**	0.40	0.18	**0.022**
3	*ZBTB20*	rs1219190601	T	G	intron	0.012	-0.38	0.10	**2.07E-04**	2.05	1.71	0.232
3	*ZBTB20*	rs76546326	C	T	intron	0.013	0.07	0.10	0.447	2.86	1.40	**0.040**

3	*ZBTB20*	rs150020524	A	G	intron	0.024	-0.01	0.07	0.851	-2.13	0.70	**2.37E-03**
3	*ZBTB20*	rs77867745	A	G	intron	0.038	-0.04	0.06	0.536	0.94	0.47	**0.043**
3	*ZBTB20*	rs116180437	T	A	intron	0.048	-0.05	0.05	0.345	-0.99	0.34	**3.29E-03**
3	*ZBTB20*	rs145272406	C	T	intron	0.015	-0.12	0.09	0.177	-4.27	0.99	**1.59E-05**
3	*ZBTB20*	rs114295131	A	C	intron	0.015	-0.12	0.09	0.174	-4.27	0.99	**1.59E-05**
3	*ZBTB20*	rs77949732	T	A	intron	0.015	-0.11	0.09	0.222	-4.27	0.99	**1.59E-05**
3	*ZBTB20*	rs146425021	A	G	intron	0.029	-0.04	0.07	0.542	-1.33	0.65	**0.039**
3	*ZBTB20*	rs201716537	C	T	intron	0.010	0.08	0.11	0.474	-3.76	1.40	**7.28E-03**
3	*ZBTB20*	rs145607299	T	C	intron	0.011	0.07	0.10	0.497	-3.51	1.21	**3.82E-03**
3	*ZBTB20*	rs931723	C	G	intron	0.204	0.08	0.03	**0.012**	-0.06	0.08	0.428
3	*ZBTB20*	rs4146802	A	C	intron	0.022	0.02	0.07	0.797	-1.57	0.63	**0.012**
3	*ZBTB20*	rs138639994	T	G	intron	0.016	-0.10	0.09	0.261	-1.46	0.73	**0.047**
3	*ZBTB20*	rs9827199	T	C	intron	0.359	0.05	0.03	0.107	0.11	0.05	**0.018**
3	*ZBTB20*	rs143323356	A	G	intron	0.046	-0.11	0.05	**0.034**	0.15	0.36	0.681
3	*ZBTB20*	rs10222496	A	C	intron	0.404	0.02	0.03	0.525	0.11	0.04	**7.86E-03**
3	*ZBTB20*	rs73228389	G	C	intron	0.408	0.02	0.03	0.516	0.12	0.04	**4.92E-03**
3	*ZBTB20*	rs144357867	G	A	intron	0.016	-0.10	0.09	0.242	-2.65	1.08	**0.014**
3	*ZBTB20*	rs7643617	A	C	intron	0.406	0.03	0.03	0.430	0.11	0.04	**9.38E-03**
3	*ZBTB20*	rs1473580	C	G	intron	0.413	0.03	0.03	0.406	0.09	0.04	**0.034**
3	*ZBTB20*	rs9851136	A	C	intron	0.418	0.02	0.03	0.483	0.09	0.04	**0.021**
3	*ZBTB20*	rs73230612	C	T	intron	0.413	0.02	0.03	0.446	0.09	0.04	**0.023**

A1, minor allele; A2, major allele; CHR, chromosome; BETA, Beta coefficients; MAF, minor allele frequency; MMSE, Mini-Mental State Examination; SE, standard error.
Analysis was obtained after adjustment for covariates including age, gender, education, physical activity, smoking, alcohol drinking, and chronic conditions.
P values of < 0.05 are shown in bold.

Supplementary Table 4. Linear regression models of associations between the MMSE scores and 11 SNPs in the *DRD1* locus, which have an evidence of association (P < 0.05).

CHR	Gene	SNP	A1	A2	Region	MAF	Dominant model			Recessive model		
							BETA	SE	P	BETA	SE	P
5	*ARL2BPP6-DRD1*	rs76373994	G	A	Intergenic	0.030	-0.16	0.07	**0.017**	-0.66	0.45	0.143
5	*ARL2BPP6-DRD1*	rs2253357	C	T	Intergenic	0.133	-0.07	0.04	**0.041**	-0.03	0.12	0.820
5	*ARL2BPP6-DRD1*	rs188263122	C	T	Intergenic	0.016	0.06	0.09	0.503	-2.84	0.92	**1.92E-03**
5	*DRD1*	rs686	G	A	3' UTR	0.152	-0.07	0.03	**0.040**	-0.07	0.10	0.522
5	*DRD1*	rs4532	C	T	3' UTR	0.152	-0.07	0.03	**0.039**	-0.07	0.10	0.474
5	*DRD1*	rs265981	A	G	3' UTR	0.145	-0.08	0.03	**0.020**	-0.08	0.11	0.459
5	*DRD1*	rs267410	A	C	2KB upstream	0.478	0.03	0.03	0.372	0.09	0.04	**0.018**
5	*DRD1-SFXN1*	rs183458028	A	C	Intergenic	0.016	0.09	0.09	0.295	-1.62	0.70	**0.020**
5	*DRD1-SFXN1*	rs267416	C	T	Intergenic	0.447	0.03	0.03	0.437	0.09	0.04	**0.023**
5	*DRD1-SFXN1*	rs2644644	G	C	Intergenic	0.451	0.01	0.03	0.855	0.08	0.04	**0.042**
5	*DRD1-SFXN1*	rs147030807	G	A	Intergenic	0.011	-0.27	0.11	**9.65E-03**	-0.88	1.71	0.609

A1, minor allele; A2, major allele; CHR, chromosome; BETA, Beta coefficients; MAF, minor allele frequency; MMSE, Mini-Mental State Examination; SE, standard error.
Analysis was obtained after adjustment for covariates including age, gender, education, physical activity, smoking, alcohol drinking, and chronic conditions.
P values of < 0.05 are shown in bold.

Supplementary Table 5. Linear regression models of associations between the MMSE scores and 3 SNPs in the *DRD4* locus, which have an evidence of association (P < 0.05).

CHR	Gene	SNP	A1	A2	Region	MAF	Dominant model			Recessive model		
							BETA	SE	P	BETA	SE	P
11	*DRD4-DEAF1*	rs183494193	T	A	intergenic	0.012	-0.17	0.10	0.088	-2.36	1.09	**0.030**
11	*DRD4-DEAF1*	rs111419860	C	T	intergenic	0.394	-0.02	0.03	0.512	0.09	0.04	**0.028**
11	*DRD4-DEAF1*	rs7109899	G	A	intergenic	0.391	-0.02	0.03	0.612	0.12	0.04	**6.68E-03**

A1, minor allele; A2, major allele; CHR, chromosome, BETA, Beta coefficients; MAF, minor allele frequency; MMSE, Mini-Mental State Examination; SE, standard error.
Analysis was obtained after adjustment for covariates including age, gender, education, physical activity, smoking, alcohol drinking, and chronic conditions.
P values of < 0.05 are shown in bold.

Supplementary Table 6. Linear regression models of associations between the MMSE scores and 12 SNPs in the *DRD5-SLC2A9* locus, which have an evidence of association (P < 0.05).

CHR	Gene	SNP	A1	A2	Region	MAF	Dominant model			Recessive model		
							BETA	SE	P	BETA	SE	P
4	*SLC2A9*	rs28523967	G	A	intron	0.151	0.07	0.03	0.053	0.26	0.10	**0.011**
4	*SLC2A9*	rs2030287	T	G	intron	0.151	0.07	0.03	0.055	0.26	0.10	**0.012**
4	*SLC2A9*	rs16888725	C	T	non coding transcript	0.150	0.07	0.03	**0.046**	0.29	0.10	**4.84E-03**
4	*SLC2A9*	rs118072948	A	G	intron	0.025	-0.14	0.07	**0.047**	0.46	0.54	0.395
4	*SLC2A9*	rs36075927	A	C	intron	0.148	0.08	0.03	**0.028**	0.32	0.11	**3.08E-03**
4	*SLC2A9*	rs11946054	C	T	intron	0.083	0.05	0.04	0.193	0.39	0.19	**0.046**
4	*SLC2A9*	rs10004947	C	T	intron	0.083	0.05	0.04	0.189	0.39	0.19	**0.046**
4	*SLC2A9*	rs145231664	T	C	intron	0.020	0.16	0.08	**0.047**	0.89	0.73	0.225
4	*SLC2A9*	rs56014085	A	G	intron	0.083	0.06	0.04	0.186	0.39	0.19	**0.046**
4	*SLC2A9*	rs56038393	A	G	intron	0.065	0.00	0.05	0.937	-0.49	0.22	**0.028**
4	*SLC2A9*	rs142956580	T	C	intron	0.015	-0.19	0.09	**0.034**	1.89	1.21	0.121
4	*SLC2A9*	rs7681699	A	G	intron	0.012	-0.29	0.10	**3.75E-03**	-3.25	2.42	0.179

A1, minor allele; A2, major allele; CHR, chromosome; BETA, Beta coefficients; MAF, minor allele frequency; MMSE, Mini-Mental State Examination; SE.
Analysis was obtained after adjustment for covariates including age, gender, education, physical activity, smoking, alcohol drinking, and chronic conditions.
P values of < 0.05 are shown in bold.

Supplementary Table 7. Linkage disequilibrium (LD) among 3 SNPs including rs11214442 in *NCAM1*, rs10891485 in *NCAM1*, and rs138333675 in *TTC12*.

Chr	Gene 1	SNP 1	Gene 2	SNP 2	r2
12	*NCAM1*	rs11214442	*NCAM1*	rs10891485	0.997
12	*NCAM1*	rs11214442	*TTC12*	rs138333675	0.003
12	*NCAM1*	rs10891485	*TTC12*	rs138333675	0.003

The measure of LD is based on the squared allelic correlation r^2.

Supplementary Table 8. Linkage disequilibrium (LD) among 3 SNPs including rs145272406, rs114295131, and rs77949732 in *ZBTB20*.

Chr	Gene 1	SNP 1	Gene 2	SNP 2	r2
12	*ZBTB20*	rs145272406	*ZBTB20*	rs114295131	1.000
12	*ZBTB20*	rs145272406	*ZBTB20*	rs77949732	1.000
12	*ZBTB20*	rs114295131	*ZBTB20*	rs77949732	1.000

The measure of LD is based on the squared allelic correlation r^2.
